# Patient-Specific Haemodynamic Analysis of Virtual Grafting Strategies in Type-B Aortic Dissection: Impact of Compliance Mismatch

**DOI:** 10.1007/s13239-024-00713-6

**Published:** 2024-03-04

**Authors:** Louis Girardin, Catriona Stokes, Myat Soe Thet, Aung Ye Oo, Stavroula Balabani, Vanessa Díaz-Zuccarini

**Affiliations:** 1 Department of Mechanical Engineering, University College London, Torrington Place, London, WC1E 7JE UK; 2 Wellcome/EPSRC Centre for Interventional and Surgical Sciences (WEISS), University College London, 43-45 Foley Street, London, W1W 7TS UK; 3 Department of Cardiothoracic Surgery, St Bartholomew’s Hospital, West Smithfield, London, EC1A 7BE UK

**Keywords:** Type-B aortic dissection, CFD simulation, Dacron graft, Virtual interventions, Compliance mismatch

## Abstract

**Introduction:**

Compliance mismatch between the aortic wall and Dacron Grafts is a clinical problem concerning aortic haemodynamics and morphological degeneration. The aortic stiffness introduced by grafts can lead to an increased left ventricular (LV) afterload. This study quantifies the impact of compliance mismatch by virtually testing different Type-B aortic dissection (TBAD) surgical grafting strategies in patient-specific, compliant computational fluid dynamics (CFD) simulations.

**Materials and Methods:**

A post-operative case of TBAD was segmented from computed tomography angiography data. Three virtual surgeries were generated using different grafts; two additional cases with compliant grafts were assessed. Compliant CFD simulations were performed using a patient-specific inlet flow rate and three-element Windkessel outlet boundary conditions informed by 2D-Flow MRI data. The wall compliance was calibrated using Cine-MRI images. Pressure, wall shear stress (WSS) indices and energy loss (EL) were computed.

**Results:**

Increased aortic stiffness and longer grafts increased aortic pressure and EL. Implementing a compliant graft matching the aortic compliance of the patient reduced the pulse pressure by 11% and EL by 4%. The endothelial cell activation potential (ECAP) differed the most within the aneurysm, where the maximum percentage difference between the reference case and the mid (MDA) and complete (CDA) descending aorta replacements increased by 16% and 20%, respectively.

**Conclusion:**

This study suggests that by minimising graft length and matching its compliance to the native aorta whilst aligning with surgical requirements, the risk of LV hypertrophy may be reduced. This provides evidence that compliance-matching grafts may enhance patient outcomes.

**Supplementary Information:**

The online version contains supplementary material available at 10.1007/s13239-024-00713-6.

## Introduction

Type-B Aortic Dissection (TBAD) is a cardiovascular disease involving a tear in the descending aorta. Following the initial dissection event, chronic TBADs exhibit a survival rate of 91%, in which 60% of patients develop late aneurysmal dilation, necessitating surgery in 25–50% of these cases [1, 2]. The merits of thoracic endovascular aortic repair (TEVAR) versus open surgery (OS) are nuanced and subject to debate [3]. In one study of 15,000 patients, TEVAR showed superior early outcomes but worse long-term survival than OS [4]. A review of 19 studies reveals an 11.1% mortality rate for chronic TBAD OS, compared with a 7.5% mortality rate for endovascular interventions [5]. OS is necessary when the dissection is complex, unsuitable for endovascular treatment, or due to tissue disorders which compromise the endovascular landing zones [6]. OS involves aortic clamping, excision, and replacement with a Dacron graft via thoraco-phreno-laparotomy. However, OS yields poor outcomes [7], such as spinal cord injury and stroke, and long-term adverse effects, such as retrograde left ventricular (LV) hypertrophy and new antegrade aortic tears [8].

Dacron grafts are significantly stiffer than native aortic tissue. The compliance mismatch between the rigid graft and the residual aorta can increase local aortic pressures and LV afterload [9, 10]. The rigidity and geometry of the graft also introduce an impedance mismatch with the native aorta, possibly causing pulse wave reflections and further local pressure elevation [11, 12]. Impedance and compliance mismatch can also increase energy loss (EL) [13]. EL is defined as the amount of energy used during the stretching of the aorta during systole and the release of the stored energy during diastole [14]. EL increases with graft stiffness, potentially resulting in increased LV mass and hypertrophy [15, 16]. Increased aortic stiffness and pressure can increase pulse wave velocity (PWV) [17], which has been associated with increased cardiovascular risks, including the risk of stroke [18]. The chosen graft dimensions are thus likely to impact patient outcomes, necessitating a careful balance of aortic compliance and graft length for optimal results.

Pre- and post-surgery non-invasive clinical measurements may guide surgery decision-making by providing metrics potentially influencing physiological mechanisms. Blood pressure is a clinical metric of key importance as high systolic brachial pressures (>140 mmHg) are linked with an increased risk of aortic dissection and re-dissection and graft failure (AD) [7, 19]. 2D-Flow MRI and 4D-Flow MRI are used to measure aortic blood velocity, while Cine-MRI measures wall displacement [20]. Flow-MRIs allow quantifying the success of reinstating the blood flow and the impact of the graft suture on proximal and distal aortic wall displacement after surgery. Wall shear stress (WSS) and pressure are known to impact vessel wall structure and aortic degeneration [2, 20]. However, the near wall resolution of 4D-Flow MRI is too coarse to accurately calculate WSS indices [21]. Using MRI data to inform patient-specific Computational Fluid Dynamics (CFD) simulations can enhance *in vivo* imaging due to its higher spatio-temporal resolution compared with MRI [22, 23]. Furthermore, CFD allows for virtual testing of surgical procedures and device sizing, which are impractical during surgery [24, 25].

Employing CFD in the context of surgical interventions for AD poses several challenges. The native aortic tissue expands and contracts with changes in blood flow and pressure [26], while the graft is relatively rigid. These contrasting structural properties substantially impact blood flow and may cause damage to tissues [8]. Thus, neglecting wall compliance and the compliance mismatch impact [27, 28] between native tissue and graft may lead to inaccurate simulation results. While a rigid wall assumption simplifies the modelling framework, compliant wall simulations predict WSS more accurately [29, 30]. Fluid-structure interaction (FSI) is commonly applied to model aortic wall compliance but has several limitations [31, 32]. Firstly, FSI relies on patient-specific aortic wall properties, which cannot be directly measured *in vivo* and vary significantly from patient to patient. As a result homogeneous wall mechanical properties taken from literature are often applied, leading to inaccurate wall movement [33]. Secondly, FSI simulations tend to be much more costly than traditional CFD approaches and relatively complex to implement. Nevertheless, investigating the effect of TBAD OS on LV afterload requires a compliant wall simulation method. To the best of our knowledge only one previous study has explored this using FSI [10] and reported that the LV mass increased after TEVAR due to LV hypertrophy and aortic stiffening.

In the present study, we employ the MBM [33], an efficient and patient-specific alternative to FSI, eliminating the need for explicit structural modelling of the aortic wall and its associated assumptions. Our study leverages the MBM to account for graft and aortic wall compliance in patient-specific CFD simulations of TBAD. This work provides insight into the clinical significance of graft length and aortic compliance mismatch in the context of OS for TBAD. Routine patient-specific medical imaging, including CT angiography, time-resolved Cine-MRI and 2D-Flow MRI, is used to inform MBM-CFD simulations. Three surgical grafting scenarios with varying graft length and compliance were used to study the impact of these parameters on aortic haemodynamics, including WSS, EL and LV afterload.

## Materials and Methods

### Data Acquisition

**Fig. 1:**
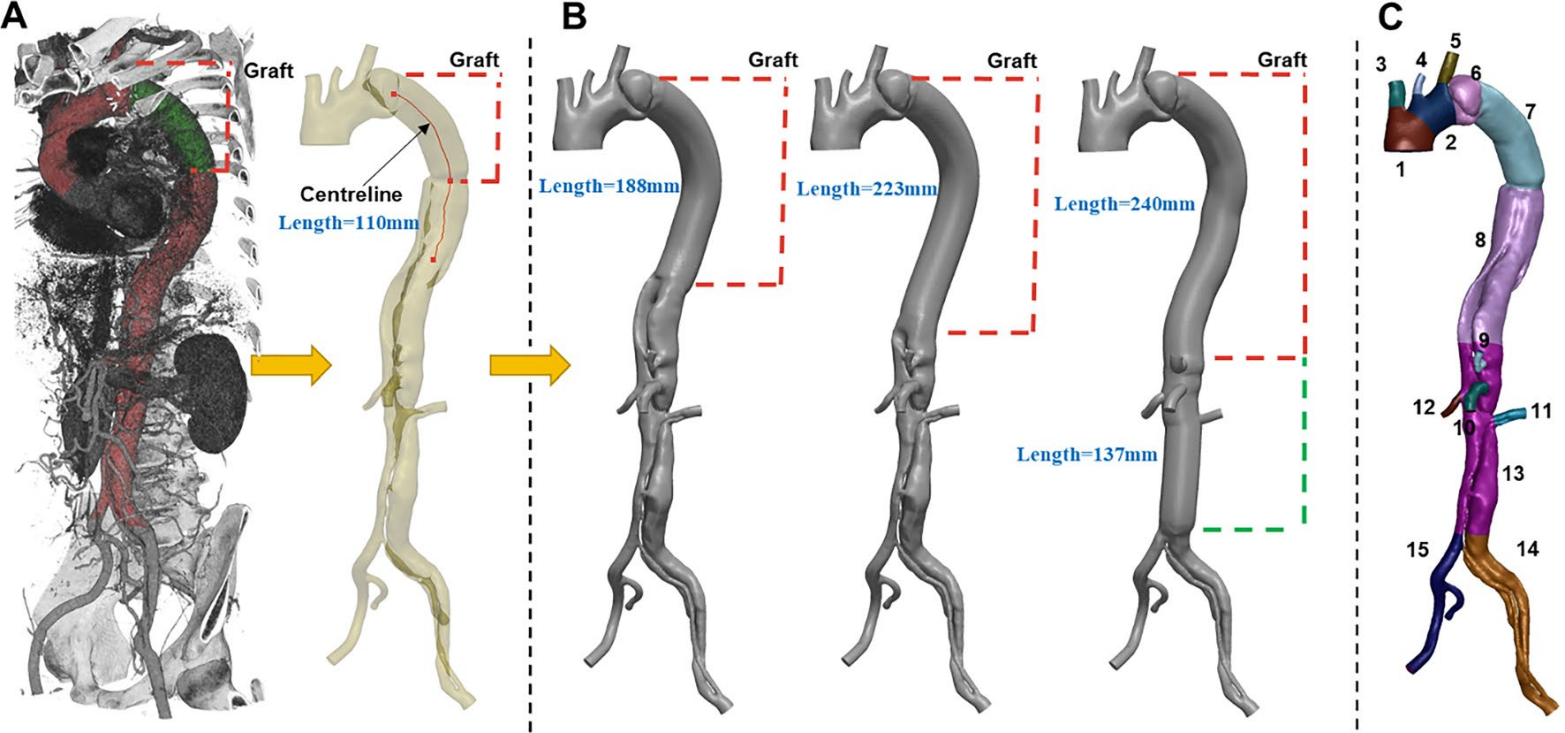
**A** Automatic 3D rendering of the CT angiography, showing, in red, the aortic vessel and, in green, the graft; the segmented post-operative geometry resulting from the CT angiography is shown next to it-namely the baseline case. **B** The three virtual surgical scenarios created from the baseline case by varying the length of graft. The red centreline from which the grafts have been swept is shown on the post-operative geometry. Red and green dashed lines indicate the extent of the 32 mm and 28 mm diameter thoracoabdominal graft of ETR, respectively. The length of each graft is indicated in blue. C aortic regions defined along the centreline using anatomical landmarks to account for proximal variations of stiffness. The numbers indicate: 1 ascending aorta, 2 arch, 3 brachiocephalic trunk, 4 left common carotid, 5 left subclavian, 6 isthmus, 7 graft, 8 descending aorta, 9 coeliac trunk, 10 superior mesenteric artery, 11 left renal, 12 right renal, 13 abdominal aorta, 14 left iliac, 15 right iliac

A patient with a complicated chronic TBAD was presented at St Bartholomew’s Hospital, London, UK. The patient underwent OS, where a dissected portion of the thoracic aorta was replaced with a graft (Gealweave, Terumo Aortic, Vascutek LTD, UK). The graft was 130 mm long with a 32 mm diameter (see Fig. 1A). Their aorta was imaged prior to and after OS. Following an ethically approved protocol (St Bartholomew’s Hospital BioResource ethical application number 97), Cine-MRI and 2D-Flow MRI were acquired pre-operatively using a Siemens MAGNETOM Aera 1.5 T (Siemens Healthcare GmbH, Erlangen, Germany) with a resolution of 1.7 mm × 1.7 mm. 2D-Flow MRI was acquired at one plane 5 cm distal to the primary entry tear (PET), located 36 mm distal to the aortic arch. CT angiography images were also acquired as part of the routine post-operative clinical examination (see Fig. 1A) using a Siemens SOMATOM Definition Edge with a resolution of 0.73 mm × 0.73 mm × 0.75 mm. Brachial pressures were acquired post-operatively. It should be noted that the patient was on medication with beta-blockers to reduce arterial pressure.

### Image Processing and Virtual Surgical Interventions

The CT angiography data (Fig. 1A) were segmented using automatic thresholding and manual correction of the mask implemented in ScanIP (Synopsis Simpleware, USA). The clinical team verified the segmented geometry, confirming the location of the tears. The resulting mask was then smoothed using Meshmixer (Autodesk, USA). The inlet and all outlets were trimmed so that their cross-sectional areas were perpendicular to the flow direction using Fluent Mesh (Ansys Fluent, USA) (Fig. 1B). Three virtual grafting scenarios were subsequently created in consultation with the clinical team by extending the graft using ScanIP and Meshmixer. Two lengths corresponding to the descending half and total length of the aorta were considered, denoted as mid-descending (MDA) and complete descending (CDA) aorta, respectively. The third grafting scenario involved an entire replacement of the thoracoabdominal aorta (ETR) to the iliac bifurcation. Two additional cases with compliant grafts were simulated, termed baseline compliant 1 (BC1), and BC2 + , described in more detail in later sections.

### Computational Mesh

Tetrahedral computational meshes were created for each domain using Fluent Mesh 19.0 (Ansys Inc., USA). Maximum and minimum cell sizes were identical across cases (4 mm, 0.35 mm). Ten prism layers with a first layer corresponding to a y+ of 1 were used to ensure appropriate boundary layer modelling for each mesh. A mesh independence study was conducted using the baseline case; coarse, medium, and fine meshes were generated by approximately doubling and dividing the maximum and minimum element sizes. The Grid Convergence Index, detailed by Craven et al. [34], was used to assess the quality of the baseline mesh. The index did not exceed 4.5% on every mesh for all metrics, consistent with past research [34]. More details are available in the Supplementary materials. Using the final mesh resolution determined from the mesh independence study, the baseline, MDA, CDA and ETR cases contained 1.35, 1.2, 1.1 and 0.9 million elements, respectively.

### Boundary Conditions

**Fig. 2:**
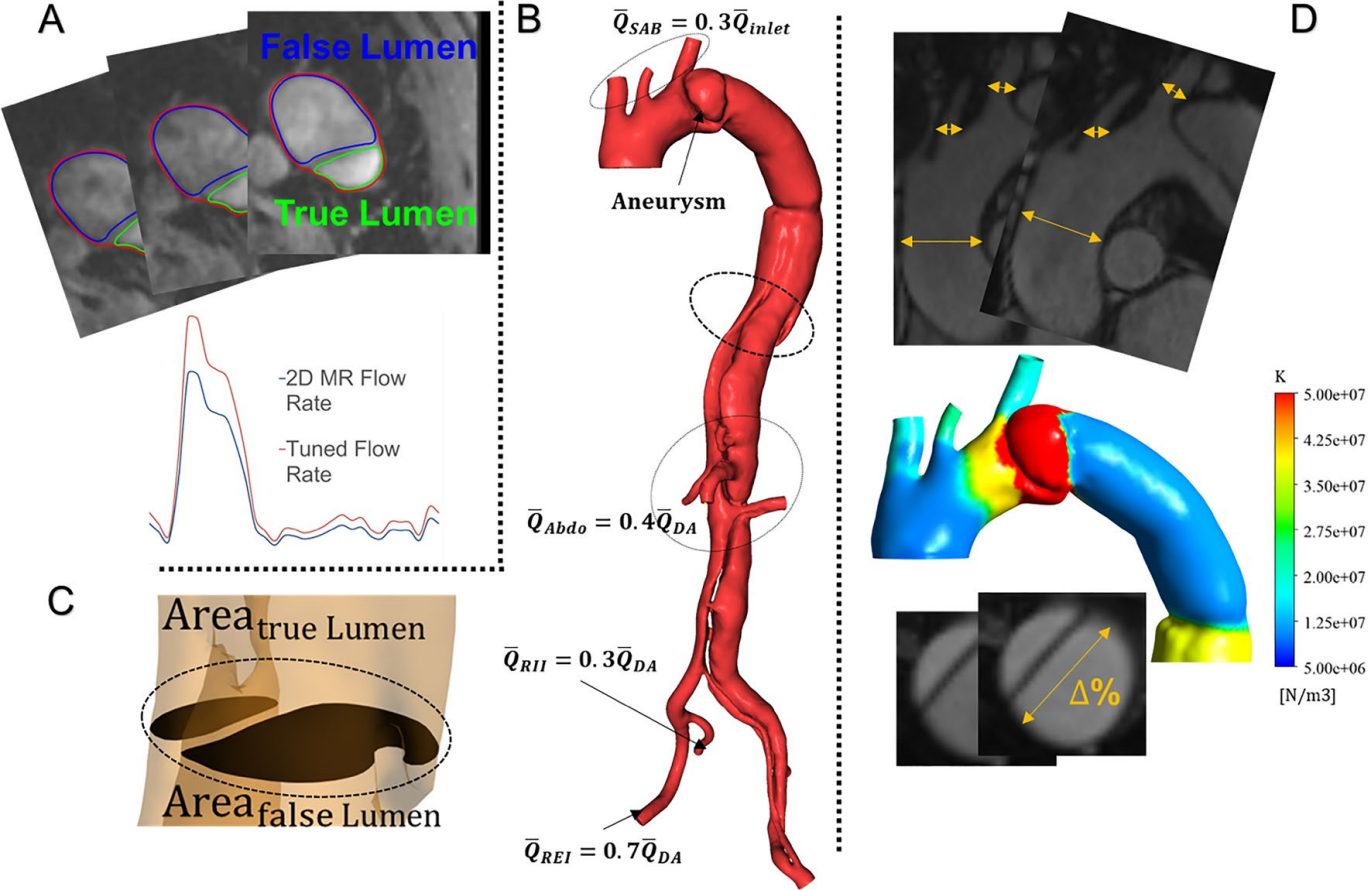
A 2D-Flow MRI plane showing in blue and green the false and true lumen respectively; below are the extracted raw and rescaled by 30% flow rates, B Flow split at the outlets: 30% of the flow leaves through the supra-aortic branches, and 40% of the remaining flow leaves through the abdominal arteries following the true and false lumen shown in C. The remaining abdominal false and true lumen flows are split as 70/30% between the exterior and interior iliac arteries; the right exterior (REI) and interior (RII) iliac arteries are shown as an example. D Sample cine-MRI planes used to measure the stiffness of the aorta. The aortic arch of BC1 is zoomed in to show the distribution of local specific stiffness values K obtained for the case of a compliant graft

The inlet flow rate was extracted from the pre-operative 2D-Flow MRI data near the aortic arch using GTFlow (GyroTools LLC., Switzerland) (Fig 2A). Considering the flow leaving the supra-aortic branches, the measured flow rate at the arch was scaled by 30% following literature values [35]. The flow rate curve was spline-interpolated in MATLAB (MathWorks Inc., USA) to match the CFD timestep of 1 ms and applied as a uniform inlet velocity profile (Fig 2A).

Three-element Windkessel (WK3) outlet pressure boundary conditions were applied to mimic the effects of the peripheral vascular system [26]. Target mean flow rates at each outlet, necessary for the calibration of the WK3 parameters [36, 37], were split as follows: 30% of the flow was assigned to the supra-aortic branches, and the mean flow rates for each branch were calculated by dividing the total supra-aortic branches flow by their respective cross-sectional area ratio, such that:

$${\overline{Q} }_{SAB,i}=0.3{\overline{Q} }_{inlet}\frac{{A}_{SAB,i}}{{A}_{tot,SAB}}$$

where ${\overline{Q} }_{inlet}$ is the mean flow rate at the inlet over a cardiac cycle (mL/s), ${A}_{SAB,i}$ is the cross-sectional area of the supra-aortic branches outlet (${m}^{2}$), and ${A}_{tot,SAB}$ the sum of the supra-aortic branches cross-sectional area (${m}^{2}$). The distribution of blood flow in the abdominal region varies among patients and can be affected by the precise nature of the dissection. A study by Amanuma et al. [38] found that the blood flow leaving the abdominal branches ranged from 25 to 75% in a group of 10 patients. After consultation with the medical team, the mean flow leaving the abdominal arteries was set as 40% of the residual flow in the descending aorta after OS. The abdominal branches are perfused by both lumens, as shown in Figure 2B, C. Hence, the mean flow rates to the abdominal branches were determined using a cross-sectional area split method, such that:

$${\overline{Q} }_{Abdo,i}=0.4{\overline{Q} }_{DA}\frac{{A}_{Abdo,i}}{{A}_{tot,Abdo}}$$

where ${\overline{Q} }_{DA}$ and ${\overline{Q} }_{Abdo}$ are the descending aorta and abdominal branches mean flow rates over a cardiac cycle (mL/s), ${A}_{Abdo,i}$ is the cross-sectional area of the abdominal branches outlet (${m}^{2}$), and ${A}_{tot,Abdo}$ the sum of the abdominal branches cross-sectional area (${m}^{2}$). The remaining mean flow was split using a 70/30% balance between the external and internal iliac arteries based on the work of Bonfanti et al. [37], as shown in Figure 2B. The same flow split methodology was applied to the four geometries and is summarised in Table 1.

**Table 1:** Mean flow rates at the outlets for each case

Qmean (mL/s)	Baseline	MDA	CDA	ETR
BT	18.5	18.5	18.5	18.5
LCC	5.1	5.1	5.1	5.1
LSA	12.1	12.1	12.1	12.1
CT	15.0	15.0	14.9	6.5
SMA	28.4	28.5	27.2	24.2
LR	12.2	12.2	11.6	11.6
RR	6.7	6.6	7.9	9.8
LEI	2.9	2.8	3.4	4.5
Lll	2.5	2.5	2.7	6.5
REI	10.2	10.2	10.1	10.2
Rll	5.7	5.7	5.7	10.2

Flow splits are very close between the post-operative, MDA and CDA cases due to close morphological similarities. Differences are found in the abdominal and iliac arteries of the ETR case due to idealised abdominal branches of the graft

Calibration of the boundary conditions using an analogue 0D model was performed to obtain the WK3 parameters following the work of Bonfanti et al. [37] and Stokes et al. [36]. The WK3 parameters obtained after calibration for the post-operative, MDA, CDA and ETR cases are presented in Table 2. The BC1 and BC2 + cases are not included in the table for clarity since the resistances are the same as those of the baseline case where the same flow split is applied to the same geometry.

**Table 2:** WK3 parameters for the Baseline, MDA, CDA and ETR cases, ${R}_{p}$ and ${R}_{d}$ are in (${\text{mmHg}}*{\text{s}}/{\text{mL}}$) , ${C}_{WK3}$ is in (${\text{mL}}/{\text{mmHg}}$)

	Baseline			MDA			FDA			ETR		
	Rp	Rd	Cwk3	Rp	Rd	Cwk3	Rp	Rd	Cwk3	Rp	Rd	Cwk3
BT	0.24	4.0	0.32	0.24	4.0	0.33	0.24	4.0	0.32	0.24	4.0	0.28
LCC	0.85	14.38	0.09	0.85	14.38	0.09	0.85	14.38	0.09	0.85	14.38	0.08
LSA	0.36	6.1	0.21	0.36	6.1	0.21	0.36	6.1	0.21	0.36	6.1	0.19
CT	0.74	12.51	0.10	0.74	12.48	0.10	0.74	12.54	0.1	0.42	7.08	0.16
SMA	0.42	7.1	0.18	0.42	7.09	0.18	0.42	7.13	0.18	0.42	7.08	0.16
LR	1.41	3.62	0.26	1.39	3.56	0.27	1.4	0.26	0.26	3.28	8.43	0.10
RR	8.45	21.74	0.04	8.54	21.95	0.04	7.95	20.44	0.05	3.28	8.44	0.10
LEI	0.15	2.48	0.5	0.15	2.48	0.51	0.15	2.61	0.47	0.17	2.94	0.37
LII	0.35	5.89	0.21	0.35	5.88	0.22	0.37	6.18	0.2	0.37	6.19	0.18
REI	0.64	10.75	0.12	0.66	11.05	0.12	0.54	9.06	0.14	0.44	7.36	0.15
RII	1.49	25.05	0.05	1.51	25.53	0.05	1.25	21.11	0.06	0.94	15.85	0.07

*Simulation of Wall Displacement and Compliant Graft Cases*

The MBM developed by Bonfanti et al. [33] was applied to simulate aortic wall displacement. The wall displacement follows the surface node normal ${\overrightarrow{n}}_{i}$ and is proportional to the difference between local and external pressures; the constant is the specific stiffness coefficient, ${K}_{i}$. The displacement ${\delta }_{i}$ of each mesh node is thus calculated as follows:

$${\delta }_{i}=\frac{{p}_{i}-{p}_{ext}}{{K}_{i}}{\overrightarrow{n}}_{i}$$

where the local pressure is ${p}_{i}$ (Pa), ${p}_{ext}$ (Pa) is the external pressure (equal to ${P}_{dia,a}$). The specific stiffness coefficient ${K}_{i}$ (N/${{\text{m}}}^{3}$) is equal to:

$${K}_{i}=\frac{2}{D}\sqrt{\frac{\pi }{{A}_{i}^{0}}}$$

where ${A}_{i}^{0}$ (${{\text{m}}}^{2}$) is the local diastolic cross-sectional area and *D* (1/Pa) is the local wall distensibility. *D* was calculated using wall movement data extracted from Cine-MRI (Fig 2D) as follows:

$$D=\frac{{A}_{max,k}-{A}_{min,k}}{{A}_{min,k}\Delta {p}_{k}}$$

where ${A}_{max,k}$ and ${A}_{min,k}$ (${{\text{m}}}^{2}$) are the maximum and minimum cross-sectional area of the aortic vessel in a given region *k* and $\Delta {p}_{k}$ is the average pulse pressure in that region*,* as estimated from a rigid, transient CFD simulation. Regions were defined along the centreline using anatomical landmarks to account for proximal variations of aortic stiffness (Fig 1C). When axial Cine-MRI images were unavailable, for example at the aortic arch, sagittal images were used to measure wall displacement. The assumption of a circular cross-section in the aorta and supra-aortic branches was employed so that diameters could be used in lieu of the cross-sectional area to calculate distensibility. The distensibility of each region was used to calculate the specific stiffness coefficient *K*, which was then mapped to its respective region of the geometry using an in-house MATLAB code. As observed in Fig 2D and following the work of Stokes et al. [36], three smoothing iterations were done to avoid discontinuities between regions of different specific stiffness. Following reported graft stiffness measurements, the graft was considered to be 20-200 times stiffer than the native aorta (K = $1.0 {10}^{9}{\text{N}}/{{\text{m}}}^{3}$) in the baseline case, MDA, CDA and ETR cases [39, 40]. Two additional cases were simulated. In the first, BC1, the graft was identical to the baseline geometry; the graft specific stiffness was equal to the measured aortic stiffness at the native ascending aorta (${K}_{BC1}=7.5{ 10}^{6}{\text{N}}/{{\text{m}}}^{3})$ (Fig 2D). The second case, BC2 + , also had an identical geometry to the baseline case but with a graft specific stiffness two times smaller than BC1 (${K}_{BC2+}=3.75{ 10}^{6}{\text{N}}/{{\text{m}}}^{3})$. This latter case aimed to simulate a graft which was more compliant than any region of the aorta.

### Computational Model

The three-dimensional, transient Navier-Stokes equations were solved using the finite-volume solver ANSYS CFX 19.0 using the Carreau-Yasuda viscosity model and empirical constants from Tomaiulo et al. [41]. Blood was modelled as an incompressible non-Newtonian fluid with a density of 1056 kg/m3. By using the Reynolds number descriptions for pulsatile blood flow in cardiovascular systems as outlined by Peacock et al. [42], determining the effective shear rate based on the research of Cagney et al. [43], and increasing the maximum velocity from the 2D-Flow MRI plane by 30% to account for supra-aortic branches flow loss, the peak ${Re}_{p}$ and critical ${Re}_{c}$ were calculated as 2257, and 3890 respectively. Under these conditions, a laminar flow assumption was used. As most aortic flows likely exhibit some degree of transitional flow, simulations assuming laminar flow were compared against Reynolds-Averaged Navier Stokes (RANS) turbulent flow simulations using the k-$\omega$ SST model. For brevity, the findings are described in the Supplementary Materials. The observed differences between laminar and turbulent flow simulations did not affect the conclusions of the study. As a result, the results reported herein are based on laminar flow simulations.

An implicit, second-order backward Euler scheme with a time step of 1 ms was used to solve the Navier-Stokes and continuity equations. During the final cycle, all equations within each timestep had a root-mean-square residual value of ${10}^{-5}$. After seven cycles, the compliant simulations reached periodic conditions, i.e., less than 1% variation in systolic and diastolic pressures between cycles. Simulations were run on the high-performance computing cluster of the UCL Computer Science Department (computational time: 23 h/cycle).

### Haemodynamic Parameters

Energy loss (EL) and WSS-driven indices were calculated in this work. EL is related to pressure and flow rate within the aorta. As a result, EL often increases in the case of AD due to increased blood pressure [44]. The heart must work harder to compensate for the increased pressure, EL, and reduced blood flow, potentially leading to heart failure [45]. The EL is calculated from the difference in the sum of static and dynamic pressures between the inlet and outlets during a cardiac cycle and is defined as follows [46]:

$$EL={TP}_{in}{Q}_{in}-\sum {TP}_{out}{Q}_{out}$$

where ${TP}_{i}={P}_{i}+0.5\rho {|\overrightarrow{{u}_{i}}|}^{2}$, $\rho$ is the blood density (${\text{kg}}/{{\text{m}}}^{3})$, ${Q}_{i}$ the volume flow rate (${{\text{m}}}^{3}/{\text{s}})$, ${P}_{i}$ the pressure (Pa), and $|\overrightarrow{{u}_{i}}|$ the velocity magnitude (m/s).

Measured as the shear force applied to the inner surface of the arteries divided by area, WSS has been linked to the development of aortic disease [47]. Three WSS-related indices are commonly employed in hemodynamic analyses: time average wall shear stress (TAWSS), oscillatory shear index (OSI), and endothelial cell activation potential (ECAP) [48]. TAWSS is the averaged WSS over a cardiac cycle and measures the total shear stress applied to the wall. OSI measures the axial directional changes of the WSS vector over the cardiac cycle. By definition, OSI varies between 0 to 0.5, indicating unidirectional WSS vector for low values and a fluctuating WSS vector for high values. ECAP is defined as the ratio of OSI and TAWSS and quantifies the degree of thrombogenic susceptibility of the aortic wall. High values of ECAP (>1.4 Pa^−1^) correspond to regions where the OSI is high and the TAWSS is small, which indicates regions susceptible to high endothelial cell deposition and thrombosis [49]. These WSS indices are as follows [50]:

$$TAWSS=\frac{1}{T}{\int }_{0}^{T}\left|\varvec{\tau} \right|dt$$

$$OSI=0.5*\left[1-\frac{\mid\int_{0}^{T}\varvec{\tau} dt\mid}{\int_{0}^{T}\mid\varvec{\tau} \mid dt}\right]$$

$$ECAP=\frac{OSI}{TAWSS}$$

where *T* is the cardiac cycle period (s), and $\tau$ the instantaneous WSS vector.

The TAWSS and ECAP differences between the baseline and the five cases examined are computed to better elucidate the impact of the various grafts on hemodynamics. The latter are normalised by the baseline average as:

$${TAWSS}_{diff}=\frac{{(TAWSS}_{baseline}^{i}-{TAWSS}_{case}^{i})}{{\overline{TAWSS} }_{baseline}}$$

$${ECAP}_{diff}=\frac{{(ECAP}_{baseline}^{i}-{ECAP}_{case}^{i})}{{\overline{ECAP} }_{baseline}}$$

## Results

### Comparisons Between Base Baseline Case CFD Results and Targeted Clinical Data

Validation and verification were performed via qualitative and quantitative comparisons between the CFD simulations of the baseline case and the target values from clinical data. The relative signed error on the metrics of interest was calculated below and shown in Table 3:

**Table 3:** Systolic ${P}_{sys,a}$ and diastolic ${P}_{dia,a}$ pressures, mean flow rate ${Q}_{mean}$ at the registered plane location and maximum diameter variation at regions of interest for the baseline simulation and clinical data measurements

	Psys (mmHg)	Pdia (mmHg)	Qmean (mL/s)	Diameter variation (mm)			
				Ascending aorta	Brachiocephalic trunk	Left common carotid	Left Subclavian
Target	96.52	68.0	83.5	1.30	0.70	0.50	0.50
Baseline	97.5	68.38	84.9	1.26	0.69	0.49	0.49
Relative error	− 1.0%	− 0.6%	− 1.6%	3.2%	1.4%	2.0%	2.0%

$$\frac{{Metric}_{Baseline}-{Metric}_{case}}{{Metric}_{case}}$$

${P}_{sys,a}$ and ${P}_{dia,a}$ were obtained within 1% of relative signed error against the brachial pressure cuff measurements. The simulated aortic wall displacement was verified against the Cine-MRI measurements; the maximum diameter variation over a cardiac cycle was measured. Measurements were taken at the AA and supra-aortic branches where most displacement occurs (Fig 2D); relative signed errors between the Cine-MRI and baseline measurements were under 2%. The coordinates of the 2D-Flow MRI plane were extracted and registered onto the CFD domain to compare the mean flow at the same location. The relative signed error between the mean flow rates was 1.6% (Fig 2A). As errors remained minor (i.e., 3.2%) between the CFD simulation and the clinical data measurements, the simulation settings were deemed suitable to be applied to the additional intervention cases.

#### Pressure, Wall Displacement and Energy Loss (EL)

**Fig 3:**
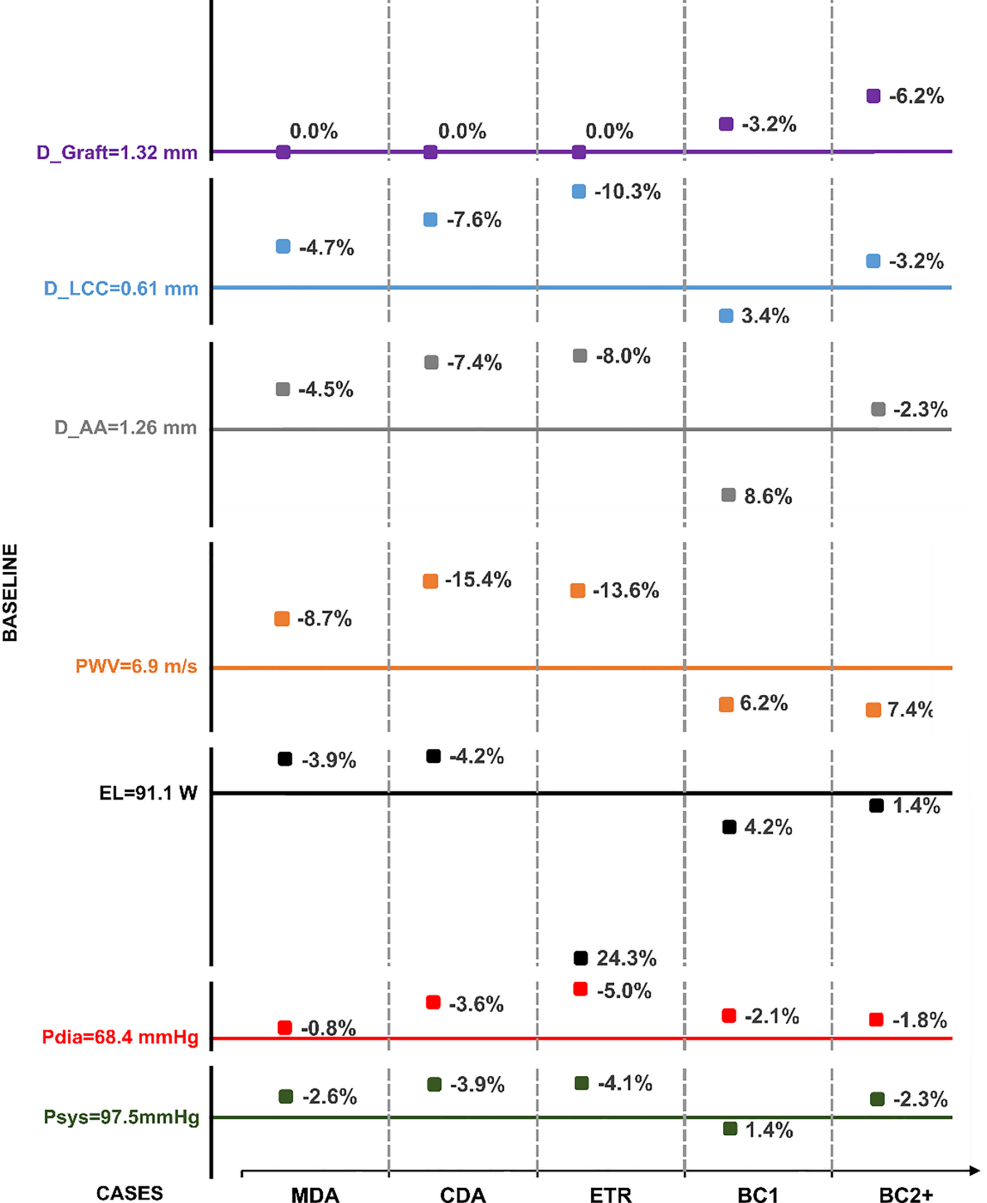
Y-axis represents systolic and diastolic pressures (Psys, Pdia), EL, PWV, and maximum diameter variation at the ascending aorta (D_AA), left common carotid (D_LCC), and graft (D_Graft) for the baseline case. The X-axis shows values of the metrics of interest for five additional cases, each labelled with their respective relative signed errors. Bold lines denote the metric values for the baseline case and those corresponding to 0% relative signed errors

The LV pressure has been reported to vary linearly with ascending aorta pressure [51]; if the ascending aorta pressure increases, so does the LV pressure. We report the ${P}_{sys,a}$ and ${P}_{dia,a}$ at the inlet of the baseline case (Fig 3) and the relative signed error with respect to the five additional cases to show the impact of graft length and compliance in pressure values (Fig 3). Inlet pressure increased with longer grafts. The ETR case had the highest systolic and diastolic pressures, however, the increasing pressure trend with graft length was not evident, as there was no significant pressure increase compared to CDA. The compliant graft used in BC1 reduced the inlet pressure; however, the trend was not linear as the pressure of BC2 + increased compared to BC1.

The PWV between the pressure peak at the inlet and the celiac trunk was calculated from the temporal difference in pressure wave peaks at a proximal and distal location in each case (Fig 3). The PWV increased by up to 15.4% in the cases where the graft was more rigid and decreased up to 7.4% in BC2 + , the case with the most compliant graft.

The maximum diameter variation of the ascending aorta, left common carotid, and graft was compared between the baseline and the five additional virtual intervention cases (for clarity purposes, only the left common carotid are shown in Fig 3 as the displacements of the two other supra-aortic branches followed the same trend). The maximum diameter variation increased along with the pressures in the rigid graft cases, with maximum values found in the ETR case. The maximum diameter of the AA and supra-aortic branches was reduced in the BC1 case, while the diameter of the compliant graft expanded by 3%. Maximum diameter variation at the three locations of interest of the BC2 + case was all larger than the baseline case; the compliant graft in BC2 + expanded by 6%.

The maximum increase in EL between the inlet and the outlets was observed in the CDA, while EL drastically decreased in the ETR case (Fig 3). EL was also slightly reduced in the BC1 case, while the change was negligible in BC2 + .

### WSS-Based Indices

**Fig 4:**
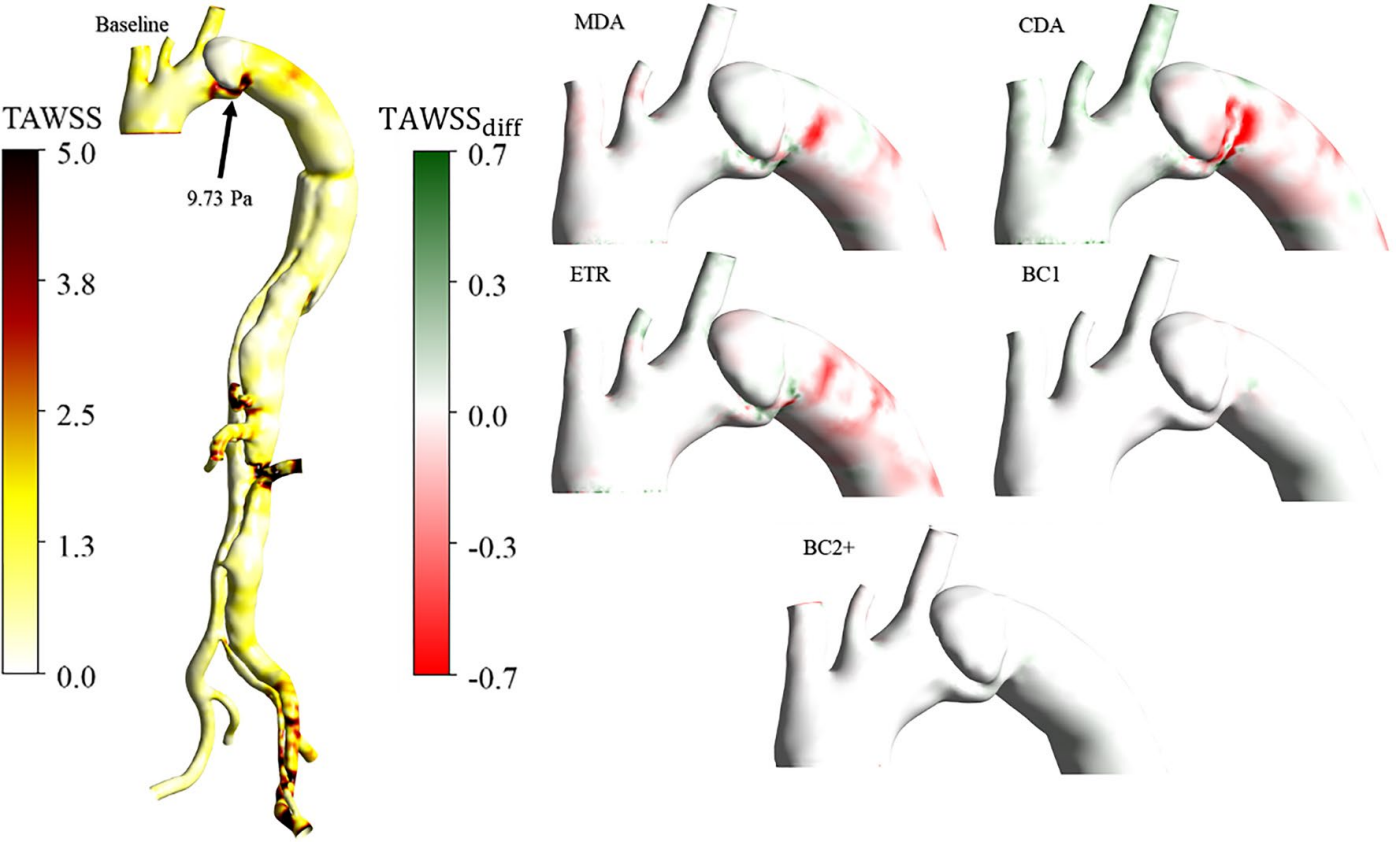
Front view of the TAWSS. On the left, values over 5 Pa are found at the PET, the abdominal arteries, and the left iliac of the baseline case. The black arrow indicates the maximum TAWSS at the PET. On the right, the TAWSS differences between the baseline and the five cases are shown. A zoom is made on the AA and aortic arch as regions of interest where the TAWSS is high

**Fig 5:**
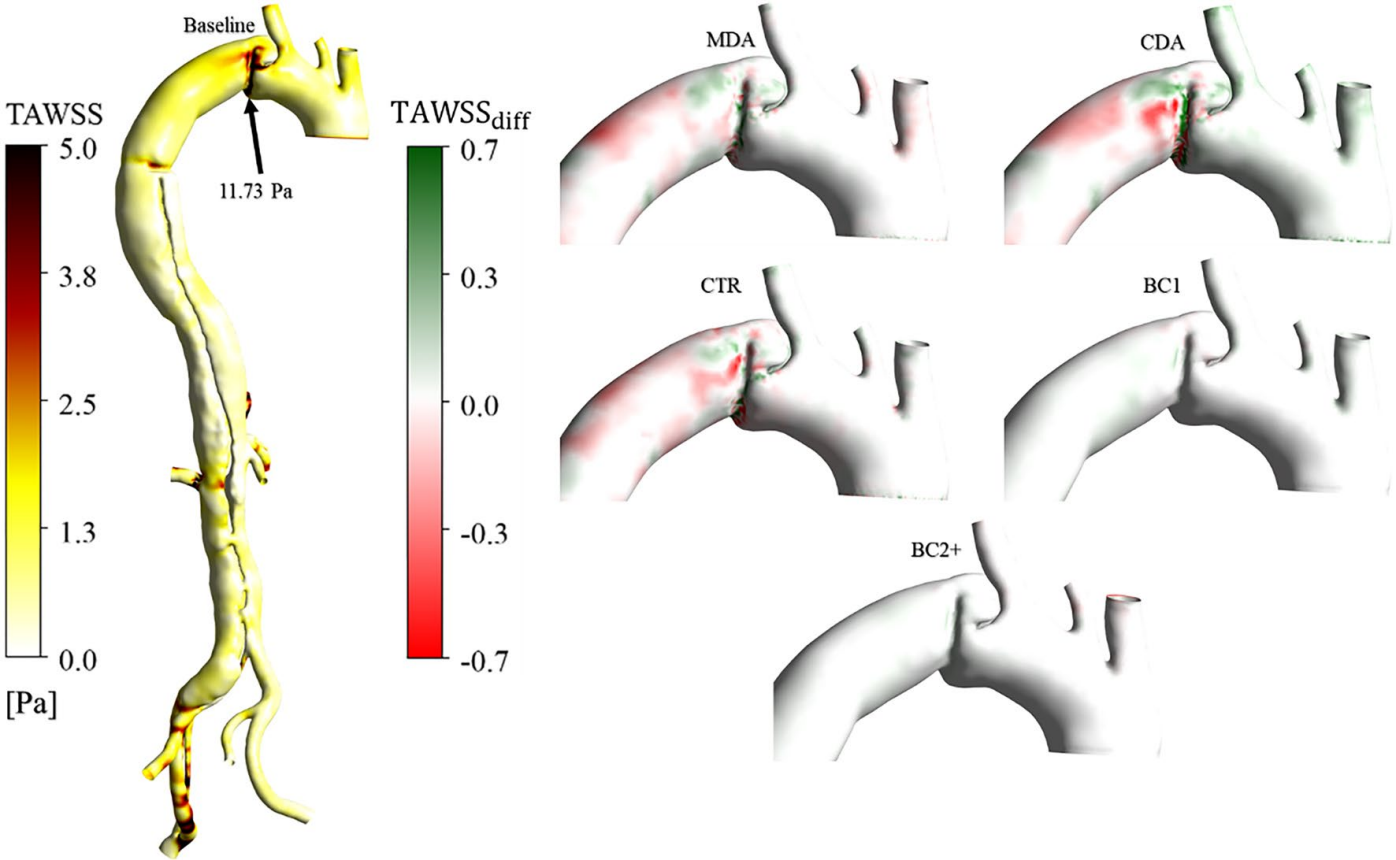
Front view of the TAWSS. On the left, values over 5 Pa are found at the PET and sutures with the graft of the baseline case. The black arrow indicates the maximum TAWSS at the PET. On the right, the TAWSS differences between the baseline and the five other cases are shown. A zoom is made on the AA and aortic arch as regions of interest where the TAWSS is high

**Fig 6:**
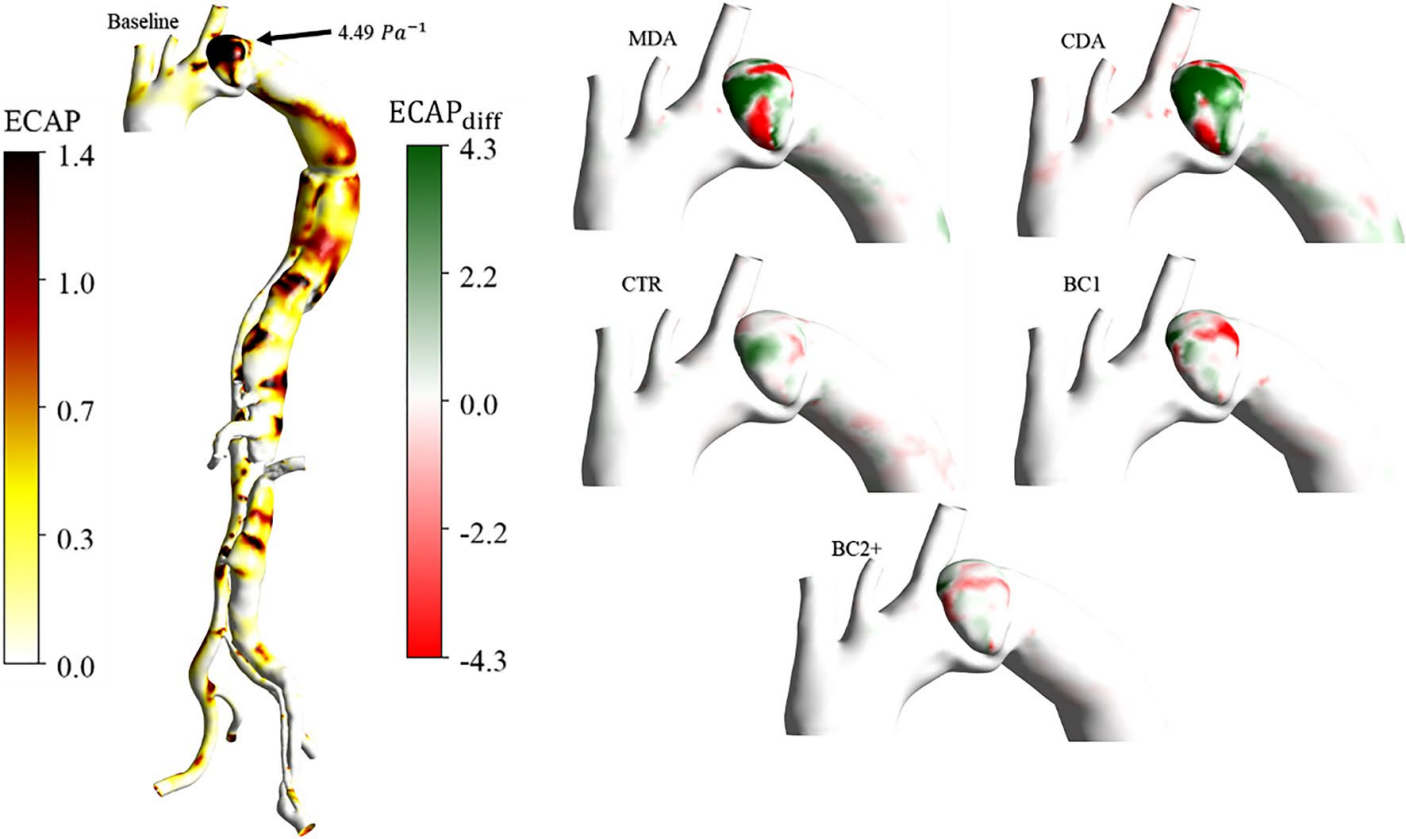
ECAP distributions, front view. On the left, ECAP absolute values for baseline case; values over 1.4 ${{\text{Pa}}}^{-1}$ are noted in the aneurysm. The black arrow indicates the maximum ECAP value in the aneurysm. On the right, ECAP differences between the baseline and the five virtual cases

Contours of TAWSS and ECAP, capped between the critical ranges (0–5 Pa) and (0–1.4 Pa^-1^), respectively, according to the literature [47, 52] are plotted in Figs. 4, 5 and 6. TAWSS distributions are similar among all cases, as the same inlet condition was applied, and the geometries are similar. The PET, aneurysm and graft sutures are the main clinical regions of interest and hence differences in estimated indices between cases are illustrated therein. For completeness, the BC1 and BC2 + cases are left on the different figures even if differences are negligible.

High TAWSS is observed in the vicinity of PET, graft sutures, and abdominal and iliac arteries in every case due to high velocities in these locations, as observed in other studies [53–55]. Differences are primarily observed at the PET and graft sutures where TAWSS is high. The TAWSS maximum values at locations of interest are indicated on the baseline case in Figs. 4 and 5 by a black arrow. TAWSS marginally increases at the sutures and PET with a longer graft minimum and maximum differences are − 0.8 and − 1.37% at the PET and 1.87% and 2.28% at the sutures for the MDA and CDA cases, respectively. TAWSS distributions were qualitatively similar between the baseline and the ETR case. Normalised differences in BC1 and BC2 + were insignificant as these cases share identical boundary conditions and geometries.

ECAP values vary mainly within the aneurysm. They are generally over the critical value of 1.4 ${{\text{Pa}}}^{-1}$ [56] with a maximum baseline value of 4.49 ${Pa}^{-1}$ , indicated by a black arrow in Fig 6. Critical ECAP values are also observed around the multiple re-entry tears proximal to the abdominal branches and the narrowing of the aortic lumens. Maximum differences in ECAP varied between 16% and 20% in the MDA and CDA cases, respectively and were much smaller, within 7% in the ETR case. Similarly, to TAWSS distributions, ECAP distributions were very similar in BC1, BC2 + and baseline cases.

## Discussion

This study examined how different grafting strategies can influence pressure, wall displacement, EL, PWV and WSS-related metrics due to compliance and impedance mismatch in a patient-specific setting.

### Pressure, Wall Displacement, PWV and EL

Longer grafts induced pressure increases up to 4% in the MDA and CDA cases. Additionally, aortic wall displacements, attributed to the pressure-related nature of the MBM, were also higher compared to the baseline case. As a result of rigid grafts, studies by Rong et al. [57] and Nauta et al. [58] found increasing diameters in supra-aortic branches after ascending and thoracic repairs. They noted increased pulse pressure and deformation of the AA and aortic arch, increasing the risk of dissection propagation or aneurysmal degeneration.

Consequently, with the stiffening added in the MDA and CDA cases, along with the increase in pressure and the impedance mismatch, the measured PWV was also higher by more than 6% (Fig 3) and EL increased by up to 4%. These two simulations suggest that grafting length impacts cardiovascular health, including its impact on the LV load. LV hypertrophy has been shown to be caused by aortic stiffening and pressure increase [59], as well as increased PWV [60]. Similarly, Qiao et al. [61] reported that the interaction between the implanted graft and wall movement may be responsible for increased EL. In an FSI study comparing a pre-and post-TEVAR case of TBAD, van Bakel et al. [10] demonstrated an increase in LV stroke work after the intervention. They concluded that the increased aortic impedance and decreased aortic compliance between the endovascular stent and the aorta led to an increased LV afterload and suggested using compliant devices.

Despite utilising the longest graft and having the lowest aortic compliance among all cases, the ETR case did not exhibit the expected increase in PWV and EL after the trend observed in the MDA and CDA cases. Pressures increased and were also the highest among the cases studied. Intrinsically, aortic wall displacements were also the highest. However, the PWV did not increase, and there was a substantial 24% reduction in energy loss (Fig. 3). The idealised geometry of the graft could explain this. The abdominal portion features four circular outlets, and the idealised geometry of the graft makes the aorta more similar to a healthy one. This likely reduces the reflection of pressure waves. If no other factors are considered, the ETR case would offer a favourable surgical option. That said, complete replacement of the aorta has been associated with serious negative consequences, such as spinal cord injury resulting in paraplegia, as most segmental arteries are no longer attached to the aorta [62]. Additionally, in the case of a more extensive dissection, kidneys must cope with an abnormal level of perfusion. Therefore, recovering a physiologically typical flow split after surgery may lead to the deterioration of renal function [63].

Moreover, the simulations of the compliant graft cases were more complex to analyse, and direct conclusions were challenging to reach. In the BC1 case (patient-specific compliant case), all metrics showed improvement compared to the baseline case. This can be attributed to the increased aortic compliance, providing an additional buffering effect that reduces pressures and wall displacement as the graft extends. The PWV was also smaller, and EL was decreased by 4% (Fig. 3). This could suggest that a patient-specific compliance-matching graft might mitigate the risk of LV hypertrophy [64]. However, such a conclusion was not readily attainable with the BC2 + (very compliant graft) case. With the graft being twice as compliant as the native proximal vessel, a compliance mismatch was also introduced. Pulse pressure increased by 3.6% compared to the baseline case, and even though the graft expanded by 6% in diameter, other regions of interest exhibited larger diameters than the baseline case (Fig. 3). This indicates a graft that is too compliant can be detrimental as it will increase aortic impedance. The implication is that the compliance mismatch between the graft and the aorta works in two ways: a graft more compliant than the natural aorta similarly increases pressure to a rigid one. Conversely, in BC2 + the PWV was reduced the most out of all cases by 7% (Fig 3), and the EL was similar to that of the baseline case, thus increasing the LV afterload. This PWV disparity reflects different grafting strategies; theoretically, greater aortic compliance results in a smaller PWV due to the damping effect of the graft. However, a highly compliant graft might induce excessive pressure reflection and an increased pulse pressure. Therefore, while increased compliance generally facilitates smoother pressure wave propagation and reduces cardiac workload, achieving a balanced approach is sensible.

In Fig. 3, a comprehensive comparison of all cases is presented, revealing that the 'best grafting' strategy aligns with a graft exhibiting native compliance similar to that of the aorta. In instances where the rigid graft scenario was simulated, and considering all indices, it becomes apparent that an ETR surgical strategy surpasses alternative surgical approaches. However, it is crucial to acknowledge the oversimplification inherent in this observation, as the considerable risk of malperfusion in patients undergoing extensive aortic replacement is well-established. Furthermore, it is emphasized that a nuanced evaluation through patient-specific CFD analysis is indispensable for exploring diverse surgical options. Nevertheless, these findings should be interpreted carefully in light of existing clinical evidence and the unique condition of the individual patient.

### WSS-Based Indices

High TAWSS has been linked with aortic wall degeneration and rupture [65] and is commonly found in narrowed regions such as the PET and re-entry tears due to higher velocity gradients in these regions [37]. High ECAP may indicate regions with an elevated risk of atherosclerotic plaque formation and calcification, a known risk factor for aortic rupture commonly found in TBAD [66]. Our results showed qualitatively similar TAWSS and ECAP distributions across all cases.

The largest normalised differences were found between the baseline and MDA and CDA cases near the PET and graft sutures, indicating a higher risk of aortic growth, dissection, and tear expansion [67]. Noticeable differences in ECAP were also observed in the aneurysmal region, with maximum increases of 16% and 20%, respectively, suggesting that graft length may influence aortic wall remodelling and disease progression.

Similar WSS-indices distributions were observed in the ETR, BC1, and BC2 + cases. Alimohammadi et al. [29] reported that in a chronic dissection where wall displacements were small, regions of high TAWSS, did not differ much between rigid and compliant wall simulations. Similarly, our results show that normalised relative differences between the baseline case, BC1 and BC2 + , were negligible.

### Compliant Biomimicking Grafts

Research has demonstrated significant progress in 3D bioprinting technology. In recent years, tissue analogues for aortic valves or blood vessels, have been successfully produced [68]. However, biomimicking technologies for compliant tissues have been mostly applied to smaller vessels [69]. Reproducing or mimicking the characteristics of the aorta remains complex and costly and has been scarcely reported [70]. Our findings suggest that compliant grafts may benefit TBAD patients after OS by reducing EL and thus reducing the risk of LV hypertrophy and heart failure. Combining *in silico* virtual grafting and *in vivo* imaging data*,* 3D bioprinting technology may facilitate further research and attract graft and stent manufacturers interest in this direction.

### Limitations

In this study, we investigated the impact of graft length and compliance in a patient-specific case of chronic TBAD using routinely acquired clinical data including limited pre-operative 2D-Flow MRI and Cine-MRI data. Using pre-operative data may introduce inaccuracies in post-intervention virtual scenarios due to changes in inlet flow rate and aortic wall compliance after the intervention. However, previous research by Pirola et al. [71] demonstrated the feasibility of using preoperative data to tune postoperative boundary conditions using post-intervention invasive aortic pressure measurements acquired during a follow-up. They showed overall acceptable agreement with their simulated post-intervention pressure. Our results suggest that this methodology can be valuable in the absence of clinical data during the follow-up of TBAD patients with grafts.

Previous studies have shown that Dacron graft stretching occurs mostly in the axial direction, with a ratio of about 50 between axial and radial stretching [39, 40, 72]. Axial stretching is approximately 20-30 times lower than the healthy ascending aorta while the radial one 40 times. The volume compliance of the graft primarily thus stems from its axial stretching; however, it was reported that the longitudinal stretching of the thoracic aorta does not exceed 1% during the cardiac cycle [73]. Once the graft is sutured at the descending aorta, its axial stretching is minimal due to its stiffness. Hence, we believe our assumption of neglecting the longitudinal displacement is valid and does not affect the simulation of a stiff graft and our conclusion.

The nonlinear and anisotropic response of the aortic tissue is complex. Without access to specific tissue in-vivo patient data, the model in this study assumes a linear relationship between pressure difference and a specific stiffness K field. This approach, adopted in our previous studies, keeps the workflow patient-specific as it uses in-vivo wall displacement data and has been extensively validated [33, 36]. Additionally, Rissland et al. [74] and Mesri et al. [75] suggest that while our model assumes a linear response, it can still provide valuable comparative conclusions. Specifically, we anticipate higher peak WSS, while the distribution of regions with low and high WSS would remain consistent, which would not change the comparisons and conclusions made between the baseline and five virtual surgical cases.

Future work will incorporate 4D-Flow MRI and Cine-MRI datasets to improve the accuracy of the simulations. These datasets will enable a more comprehensive validation of the simulations, such as using the PWV to inform the compliant model further. This approach may lead to well-validated simulations informed by rich datasets, potentially capturing key haemodynamic variables of interest more accurately.

## Conclusions

This study simulated a patient-specific post-operative case of TBAD and explored the impact of different surgical strategies via virtual grafting. Specifically, we conducted five simulations, i.e., virtual interventions, including three virtual surgeries using varying graft sizes and two cases with compliant grafts. To the author's knowledge, this study is the first investigation in the literature to evaluate the influence of various potential surgical strategies for TBAD on key haemodynamic markers, WSS distribution, and LV workload, considering the effects of graft length and compliance.

Our findings suggest that reducing aortic volume compliance by increasing the length of rigid grafts in the MDA and CDA increases pressure, PWV and EL. However, a graft with a compliance matching the natural aortic compliance of the patient lowered the inlet and pulse pressures and EL. Cases such as the ETR and BC2 + showed mixed performances in the metrics of interest. In conclusion, optimal graft selection cannot be determined without considering the morphology and condition of the aorta of each patient and in any case, any results should be used as a guideline and carefully considered against clinical evidence and expertise. This study illustrates that exploring various virtual grafting strategies via patient-specific simulations can aid this process. Graft manufacturers should consider developing biomimetic grafts to reduce the risk of LV hypertrophy and heart failure in future TBAD patients. However, this endeavour would have numerous challenges as it would need to mimic aortic compliance on almost a case-by-case basis.

### Supplementary Information

Below is the link to the electronic supplementary material.

Supplementary file1 (DOCX 823 KB)

## Abbreviations

AD: Aortic dissection
BC1: Baseline compliant one
BC2 +: Baseline compliant two plus
CDA: Complete descending aorta
ECAP: Endothelial cell activation potential
EL: Energy loss
ETR: Entire thoracoabdominal replacement
FSI: Fluid-structure interaction
LV: Left ventricular
MDA: Mid-descending aorta
MBM: Moving boundary method
OS: Open surgery
OSI: Oscillatory shear index
PET: Primary entry tear
PWV: Pulse wave velocity
TAWSS: Time-average wall shear stress
TBAD: Type-B aortic dissection
TEVAR: Thoracic endovascular aortic repair
WSS: Wall shear stress
