# Supplementary material for: Patient-Specific Haemodynamic Analysis of Virtual Grafting Strategies in Type-B Aortic Dissection: Impact of Compliance Mismatch
Source: Cardiovasc Eng Technol. 2024 Mar 4;15(3):290–304. doi: 10.1007/s13239-024-00713-6 (PMC11239731; doi:10.1007/s13239-024-00713-6)
Supplement: Supplementary file 1 — Supplementary file1 (DOCX 823 KB) [file 13239_2024_713_MOESM1_ESM.docx]

Patient-Specific Haemodynamic Analysis of Surgical Strategies in Type-B Aortic Dissection using Virtual Dacron Grafts

*Louis Girardin^1,2^, Catriona Stokes^1,2^, Myat Soe Thet^3^, Aung Ye Oo^3^, Stavroula Balabani^1,2^,Vanessa Díaz-Zuccarini^1,2^*

*^1^Department of Mechanical Engineering, University College London, Torrington Place, London WC1E 7JE, UK; ^2^Wellcome/EPSRC Centre for Interventional and Surgical Sciences (WEISS), University College London, 43-45 Foley Street, London W1W 7TS, UK; ^3^Department of Cardiothoracic Surgery, St Bartholomew's Hospital, West Smithfield, London EC1A 7BE*

Address correspondence to Vanessa Díaz-Zuccarini and Stavroula Balabani, Department of Mechanical Engineering, University College London, Torrington Place, London WC1E 7JE, UK.

Electronic mail: v.diaz@ucl.ac.uk

ORCID:000000280172405

## Mesh sensitivity analysis

A description of the mesh sensitivity study can be found in this section. The quality of the mesh and the analysis were assessed on six planes (Fig 1) of interest using the following metrics $f_{i}$: mean and maximum velocity and time average wall shear stress (TAWSS). In addition, the relative error between the metrics was computed between the coarse (M1) and medium (M2), and M2 and the fine (M3) meshes. Also, the grid convergence index (GCI) was computed following the study of Craven et al., [1], and the GCI was calculated as follows:

$r\sim{(\frac{N_{3}}{N_{2}})}^{1/3}\sim{(\frac{N_{3}}{N_{2}})}^{1/3}$w

$$p=\frac{ln(\frac{|f_{1}-f_{2}|}{{|f}_{2}-f_{3}|}}{ln(r)}$$

$$E_{2,1}=\frac{|f_{1}-f_{2}|}{f_{2}.{(r}^{p}-1)} E_{3,2}=\frac{|f_{2}-f_{3}|}{f_{3}.{(r}^{p}-1)}$$

$$GCI_{2,1}=F_{s}\left| E_{2} \right| GCI_{3,2}=F_{s}|E_{3}|$$

With $N_{1,2,3}$ the number of elements of M1, M2 and M3, $f_{1,2,3}$ is the evaluated metric for each mesh, $F_{S}$ is a safety factor equal to 1.25 defined by Celik et al., [2] and used by Armour et al., [3].


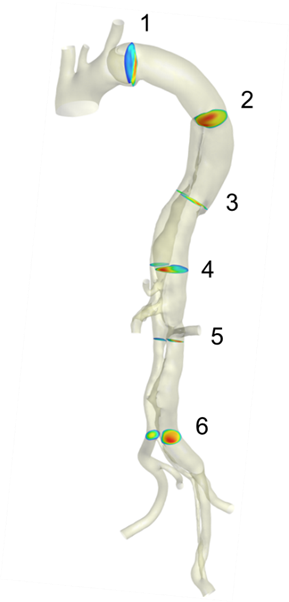


# Figure 1 Planes used for the mesh independence study.

Tables gathering all the measurements and derived metrics can be found bellow:

| Mean TAWSS | | | | | | |
| --- | --- | --- | --- | --- | --- | --- |
| Plane | 1 | 2 | 3 | 4 | 5 | 6 |
| M1 [Pa] | 0.135 | 0.178 | 0.129 | 0.115 | 0.154 | 0.145 |
| M2 [Pa] | 0.136 | 0.185 | 0.130 | 0.116 | 0.156 | 0.144 |
| M3 [Pa] | 0.139 | 0.183 | 0.130 | 0.116 | 0.156 | 0.144 |
| ${\%}_{m,c}$ | 1% | 4% | 1% | 1% | 2% | -1% |
| ${\%}_{f,m}$ | 2% | -1% | 0% | 0% | 0% | 0% |
| ${GCI}_{2,1}$ | 0.41 | 7.29 | 6.94 | 2.60 | 3.03 | 1.50 |
| ${GCI}_{3,2}$ | 0.02 | 1.64 | 3.54 | 0.64 | 0.77 | 0.37 |

# Table 1 Mean TAWSS on the wall nodes of the planes of interest. Relative error and GCI between the specified mesh

| Mean velocity | | | | | | |
| --- | --- | --- | --- | --- | --- | --- |
| Plane | 1 | 2 | 3 | 4 | 5 | 6 |
| M1 [m/s] | 0.614 | 0.685 | 0.612 | 0.427 | 0.370 | 0.313 |
| M2 [m/s] | 0.651 | 0.845 | 0.583 | 0.455 | 0.346 | 0.302 |
| M3 [m/s] | 0.653 | 0.863 | 0.598 | 0.462 | 0.352 | 0.299 |
| ${\%}_{m,c}$ | 6% | 23% | -5% | 7% | -7% | -4% |
| ${\%}_{f,m}$ | 0% | 2% | 3% | 2% | 2% | -1% |
| ${GCI}_{2,1}$ | -2.41 | 2.26 | 0.78 | 1.88 | 0.75 | 1.56 |
| ${GCI}_{3,2}$ | -4.35 | 0.79 | 0.30 | 1.34 | 0.20 | 1.04 |

# Table 2 Mean velocity on the planes of interest. Relative error and GCI between the specified mesh

| Maximum velocity | | | | | | |
| --- | --- | --- | --- | --- | --- | --- |
| Plane | 1 | 2 | 3 | 4 | 5 | 6 |
| M1 [m/s] | 1.209 | 0.5409 | 0.4775 | 0.4221 | 0.9172 | 0.6162 |
| M2 [m/s] | 1.228 | 0.5903 | 0.479 | 0.4255 | 0.9283 | 0.6232 |
| M3 [m/s] | 1.238 | 0.5811 | 0.486 | 0.431 | 0.933 | 0.6239 |
| ${\%}_{m,c}$ | 2% | 9% | 0% | 1% | 1% | 1% |
| ${\%}_{f,m}$ | 1% | -2% | 1% | 1% | 1% | 0% |
| ${GCI}_{2,1}$ | 2.15 | 2.39 | -0.50 | -2.62 | 1.10 | 0.16 |
| ${GCI}_{3,2}$ | 1.12 | 0.45 | -2.29 | -4.18 | 0.46 | 0.02 |

# Table 3 Maximum velocity on the planes of interest. Relative error and GCI between the specified mesh

## Comparison between laminar and turbulent flow simulations

In our study, the flow throughout the aorta is unlikely to be strictly laminar as transitional flow has been observed even in vessels at very low Reynolds numbers, as described by Saqr & Zidane, 2022 [4]. The assumption of using a laminar flow model was evaluated. We ran a turbulent flow simulation using the RANS model k$\omega$-SST model in CFX with 1% turbulent intensity used previously in the literature [5,6].

RANS models characteristic turbulence parameters like turbulent kinetic energy and dissipation rate through transport equations without directly resolving turbulent scales. In many simulations of aortic blood flow, the RANS 𝑘−𝜔 SST model, as presented by Lantz et al. [7], is commonly employed. However, a comparative investigation by Andersson and Karlsson, [8], scrutinising RANS and LES in the context of aortic coarctation revealed notable disparities. RANS simulations exhibited limited agreement with LES, significantly underestimating turbulence anisotropy and turbulent kinetic energy, particularly in near wall regions. Despite its limitations, the RANS model is still widely adopted for its cost-effectiveness.

This analysis compared the contours of point-wise differences of TAWSS, OSI and ECAP, and mean and max values were evaluated (Figure 2). Additionally, systolic and diastolic pressures, mean flow rates at the outlets, pulse wave velocity and energy loss were compared between the two models.

Comparing the point-wise differences of TAWSS, OSI, and ECAP, similar distributions are observed between the laminar and turbulent flow simulations. Although there is a slight shift in the distribution of high TAWSS values in the turbulent flow simulation, the overall pattern remains consistent with the laminar flow simulation. Peak TAWSS are found at the entry tear, the narrowed dissection and the right renal, indicating areas of high near-wall velocity. While there is a local maximum increase of 3.29 Pa at the right renal region in the turbulent simulation, it is important to note that the laminar values are already above 5 Pa. This suggests that, despite the shift in the peak values, the conclusions regarding the potential risks associated with elevated TAWSS (>5Pa) remain valid in the turbulent flow simulation. OSI and ECAP point-wise distributions did not exhibit any differences, which could alter the message of the paper. Additionally, systolic and diastolic pressures were 0.8 mmHg higher in the turbulent case. The turbulent flow simulation also led to a maximum relative error of 3% in mean flow rates. Energy loss and pulse wave velocity increased by less than 1%.

The differences in comparing the laminar and SST flow simulations did not affect our conclusions on these clinical metrics. Thus, we have retained the original laminar flow simulations in our study.


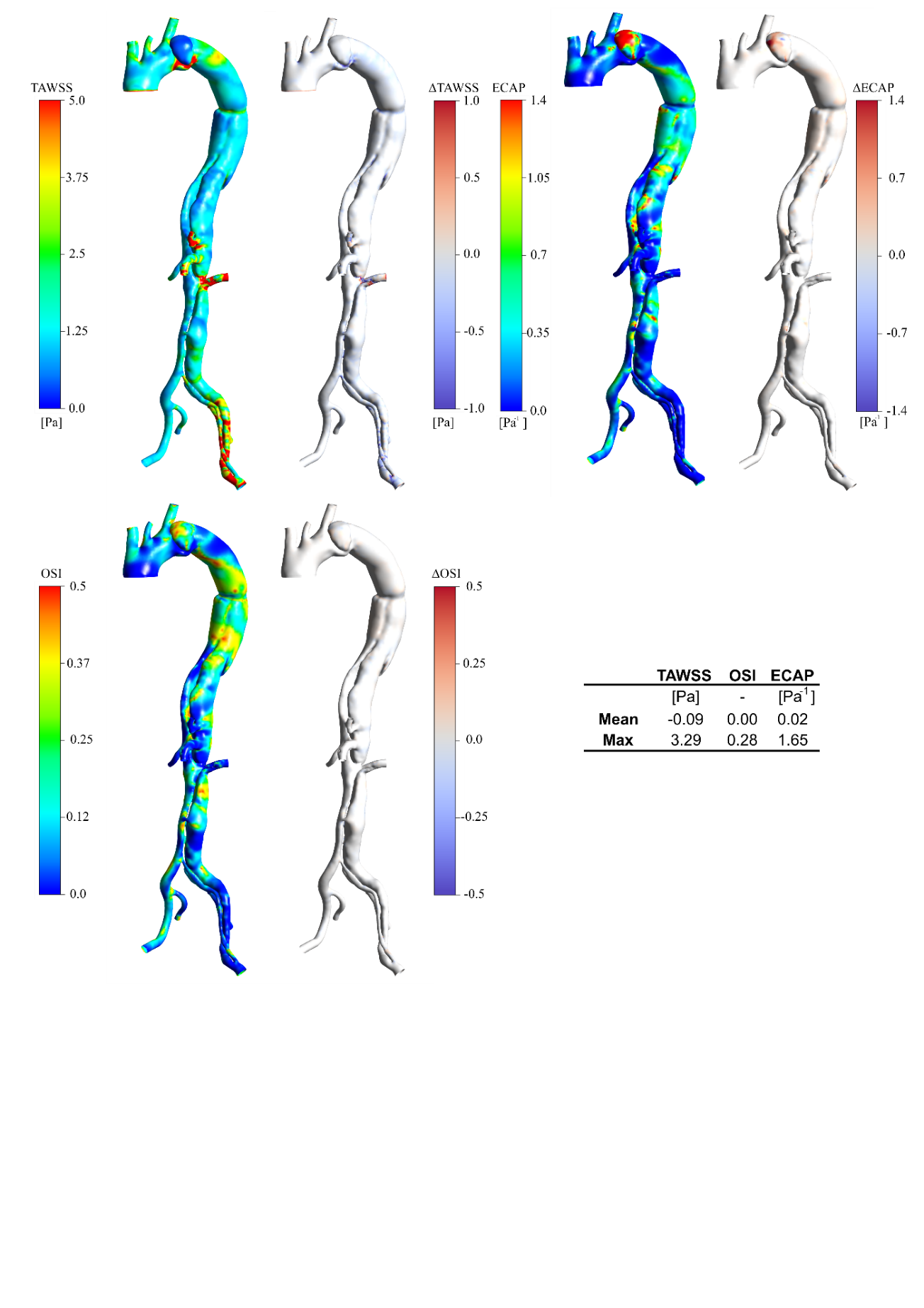


# Figure 2 TAWSS, OSI and ECAP contour map of the laminar simulation, and point-wise differences between the laminar and turbulent simulations. The table shows the mean and max point differences for TAWSS, OSI and ECAP

# Table 4 Mean flow rates at the outlets, inlet systolic and diastolic pressures, energy loss and pulse wave velocity for the laminar and turbulent simulation; the last column is the difference between the laminar and turbulent simulations

[1] B. A. Craven, E. G. Paterson, G. S. Settles, and M. J. Lawson, “Development and verification of a high-fidelity computational fluid dynamics model of canine nasal airflow,” *J Biomech Eng*, vol. 131, no. 9, pp. 1–11, 2009, doi: 10.1115/1.3148202.

[2] I. B. Celik, U. Ghia, P. J. Roache, C. J. Freitas, H. Coleman, and P. E. Raad, “Procedure for estimation and reporting of uncertainty due to discretisation in CFD applications,” *Journal of Fluids Engineering, Transactions of the ASME*, vol. 130, no. 7, pp. 0780011–0780014, 2008, doi: 10.1115/1.2960953.

[3] C. H. Armour *et al.*, “The influence of inlet velocity profile on predicted flow in type B aortic dissection,” *Biomech Model Mechanobiol*, vol. 20, no. 2, pp. 481–490, Apr. 2021, doi: 10.1007/s10237-020-01395-4.

[4] Saqr, K. M., & Zidane, I. F. (2022). On non-Kolmogorov turbulence in blood flow and its possible role in mechanobiological stimulation. *Scientific Reports*, *12*(1). https://doi.org/10.1038/s41598-022-16079-5

[5] Stokes, C., Haupt, F., Becker, D., Muthurangu, V., von Tengg-Kobligk, H., Balabani, S., & Díaz-Zuccarini, V. (2023). The Influence of Minor Aortic Branches in Patient-Specific Flow Simulations of Type-B Aortic Dissection. *Annals of Biomedical Engineering*, [doi:10.1007/s10439-023-03175-4](https://doi.org/10.1007/s10439-023-03175-4)

[6] Kousera, C. A., Wood, N. B., Seed, W. A., Torii, R., O’Regan, D., & Xu, X. Y. (2013). A numerical study of aortic flow stability and comparison with in vivo flow measurements. *Journal of Biomechanical Engineering*, *135*(1), doi:10.1115/1.4023132

[7] Lantz, J., Gårdhagen, R., & Karlsson, M. (2012). Quantifying turbulent wall shear stress in a subject specific human aorta using large eddy simulation. *Medical Engineering and Physics*, *34*(8), 1139–1148. https://doi.org/10.1016/j.medengphy.2011.12.002

[8] Andersson, M., & Karlsson, M. (2021). Characterization of anisotropic turbulence behavior in pulsatile blood flow. *Biomechanics and Modeling in Mechanobiology*, *20*(2), 491–506. https://doi.org/10.1007/s10237-020-01396-3
